# The Three Seasons of a Tropical Subsistence Farmer

**DOI:** 10.4269/ajtmh.18-0231

**Published:** 2018-06

**Authors:** Claire Panosian Dunavan

**Affiliations:** Division of Infectious Diseases; University of California; Los Angeles, CA 90095-1688; E-mail: cpanosian@mednet.ucla.edu

For many people in East Timor, a small Southeast Asian state that is profoundly food-insecure, the season is either “wet, dry, or hungry.” The same dismal prospect continues to haunt millions of farmers the world over. Will coming decades finally end this chronic cycle of want? And where does tropical medicine fit in the modern nexus of food, agriculture, and health?

In November 2017, a meeting at Stanford University provided a modern primer on rural poverty and agricultural development with a special focus on Africa. The conference featured four global leaders: 1) Agnes Kalibata, Rwanda’s former Minister of Agriculture and Animal Resources and current President of the Alliance for a Green Revolution in Africa (AGRA); 2) Kanayo Nwanze, Past President of the United Nations’ International Fund for Agricultural Development and winner of the 2016 Africa Food Prize; 3) Rajiv Shah, President of the Rockefeller Foundation and former Administrator of the United States Agency for International Development; and 4) Usha Barwale Zehr, Director and Chief Technology officer at Maharashtra, one of India’s largest multinational seed companies.

Seated next to me at Stanford was a former college classmate. An industrial engineer and entrepreneur, from 2004 to 2009 Thomas Riley also served as the United States Ambassador to Morocco, where he helped negotiate the first-ever free trade agreement between the United States and an African nation. Since then, Riley has continued to visit Africa and connect with fellow ambassadors.

Impulsively, I asked Tom for his thoughts about current funding for African development. “Too much US government investment in health, not enough in agriculture,” he replied, matter-of-factly. His comment stuck with me.

Then came the round-table and some irrefutable facts. As moderator Etharin Cousins stated at the outset: “Today some 100 million of the farmers across Sub-Saharan Africa farm less than 2 hectares of land. Eighty percent of those living in rural areas are poor. More than 30% of the rural population are chronically hungry and 35% of the under-5-year-olds are stunted.” By 2050, when the world’s population is expected to top 9 billion, Cousins (who formerly directed the UN World Food Program) added, Africa will have experienced the lion’s share of growth.

Kanayo Nwanze added a rhetorical question about a central paradox of Africa—namely, that a continent possessing two-thirds of the world’s uncultivated-but-fertile land continues to suffer devastating food insecurity. “By 2050, who is going to feed Africa? A man in his 60s? A woman with a hoe? No! Africa needs the agriculture of tomorrow, which will first feed Africa, then the world.”

Nwanze is not just good with words. Originally trained as an agricultural entomologist, early in his career, he helped with efforts to control a potentially devastating influx of mealworm attacking African cassava; as Director-General of the Africa Rice Center, he also promoted New Rice for Africa, a high-yield, drought- and pest-resistant strain specifically developed for Africa.

A day later, I ordered Nwanze’s book. Published in 2017, “*A Bucket of Water—Reflections on Sustainable Rural Development*” is a slim, thoughtful work describing many strategies that could further increase smallholder yields in Africa and beyond. The book also discusses climate change and soil degradation, civil conflict and migration, women’s role in farming, the retention of young people in agricultural enterprise, and the promise of research, technology, and innovation from gene-edited seeds to reliable electricity, internet connectivity, and satellite-based imaging (to help grow drought-resistant crops, for example) as well as improved storage and transport of crops and access to financing and insurance to accelerate uptake of new technologies. “*A Bucket of Water*” also offers lessons from Asia, where 38% of cultivated land is currently irrigated as opposed to 6% in Africa, and in South Asia, where 149 kg of fertilizer is applied per hectare of land. In Africa, the current use of fertilizer is nearly 90% lower.

Agnes Kalibata is another agricultural entomologist who has championed smallholder access to improved seeds as well as networks of “agro-shops.” First appointed Rwanda’s Minister of Agriculture in 2008, 6 years later, she moved to AGRA, an organization jointly funded by the Gates Foundation and the Rockefeller Foundation. Kalibata’s dedication to private-sector cooperatives resonates with Nwanze’s pragmatic take on farming. As he writes in *A Bucket of Water*, “Profit and income are not dirty words for poor farmers. Whether the entrepreneur is a multinational firm or a young woman selling vegetables at a roadside stand, farming is a business.”

Usha Barwale Zehr cited the legacies of the Rockefeller and Ford Foundations in bringing “good, high-yield planting material” to India and forecast future public–private partnerships in Africa focused on hybrid seeds and solar-powered water pumps. Rajiv Shah not only touted real-time data to sustain maximum productivity but predicted that Africa could someday lead the world in cultivating protein- and micronutrient-rich crops.

So, once again, what is the take-home for members of the American Society of Tropical Medicine and Hygiene? Can those fighting tropical disease share “space” and resources with experts and visionaries in global agriculture? At the very least, should not we engage around important areas of overlap?

Rajiv Shah, the only medical doctor on the Stanford dais, would likely agree. In another 2017 speech, he reiterated a core belief shared by John D. Rockefeller, Sr. and Frederick Gates, Rockefeller’s chief philanthropic advisor. “Scientific agriculture,” said Shah, “was seen (by them) as a promising way to fulfill their foundation’s mission of promoting the well-being of humanity throughout the entire world. Initially, the foundation fought disease—building schools of medicine and public health, combating yellow fever and malaria. Then after seeing millions starve during World War II, and as booming population growth threatened to outpace global food production, Rockefeller turned to fighting hunger as the greatest enemy of human well-being.”

Shah shared this story at the Norman E. Borlaug International Symposium, an annual 3-day event focused on global food security and nutrition which culminates in the presentation of the World Food Prize. Last year’s laureate was Dr. Akin Ayodeji Adesina, Nigeria’s former Minister of Agriculture and the current President of the African Development Bank; the Africa Fertilizer Summit which Adesina organized in 2006 laid groundwork for AGRA.

After AGRA was launched, former UN Secretary-General Kofi Annan served as its Chairman. Fast forward to March 2018. Writing in *Nature* about the current state of global hunger, Annan described both significant progress and patchy results since codification of the first Millennium Development Goal (to cut extreme poverty and hunger in half by 2015); continued progress, Annan argued, will require an even greater commitment to collecting granular data on vulnerable women, children, and infants, especially if the 2030 Sustainable Development targets for reduced stunting and wasting are to be met.

For another perspective on fostering more discussion between experts in agriculture and health, I turned to Dean Jamison, a global health economist and lead author of the 1993 World Bank Development Report “*Investing in Health*.” Jamison, by his own admission, has a “narrowly technocratic view of public health” as opposed to a focus on its broader determinants. But early in his career, he also worked as a World Bank education economist in China. At that time, he told me that the Chinese Communist government was already enforcing an absolute floor on food consumption, which has subsequently risen, thus assuring a certain nutritional equity.

Our conversation caused Jamison to ponder the intellectual price of hunger coupled with poor household hygiene. “(The association between) persistent low-grade diarrhea and decreased cognitive development is an especially interesting dilemma,” he said. “Why are rural kids in China so far ahead of Indians on math scores? Is it related to better school? More school? More nutrition and micronutrients? Or are the Indian kids’ minds simply on food versus quadratic equations?”

A second notable book published in 2012 does not answer that question, but what it does portray in sometimes agonizing detail is the daily burden of food insecurity as experienced by four smallholders in western Kenya. Ironically (but not surprisingly), in Roger Thurow’s “*The Last Hunger Season: A Year in an African Farm Community on the Brink of Change*,” the farmers’ principal trade-offs involve money for food versus money for seeds versus money for their children’s school fees. Hunger chronically shadowing the world’s bottom billion is seen through a different lens—at once, coolly analytical and technologically upbeat—in Sir Gordon Conway’s comprehensive text “*One Billion Hungry—Can We Feed the World?*,” a 2012 Foreign Affairs Magazine Best Book of the Year. And for those following the real-time unfolding of South–South agricultural cooperation, “*Agricultural Development and Food Security in Africa—The Impact of Chinese, Indian, and Brazilian Investments*” edited by Fantu Cheru and Renu Modi delivers a generally positive assessment of short- and mid-term results of current state- and privately funded overseas projects. But what of the long-term benefits to local communities of foreign investment in large-scale agricultural ventures? And what about land rights? As Cheru and Modi state, “The issue of land rights goes beyond policies on agricultural development. It is part and parcel of the unfinished governance agenda of Africa.”

Politics aside, speaking to fellow tropical medicine and global health specialists, the current question troubling me is this. Have we fallen short in appreciating the continuing problem of rural hunger and its many negative consequences for health and development? Perhaps, the time has come to re-assess our collective IQ regarding new initiatives in agricultural development along with global nutrition, food-borne illness, and food security.
